# Problems and Challenges to Adaptation of Gluten Free Diet by Indian Patients with Celiac Disease

**DOI:** 10.3390/nu5124869

**Published:** 2013-11-27

**Authors:** Preeti Rajpoot, Govind K. Makharia

**Affiliations:** Departments of Gastroenterology and Human Nutrition, All India Institute of Medical Sciences, New Delhi 110029, India; E-Mail: prajpoot15@gmail.com

**Keywords:** nutrition, India, wheat, barriers, compliance, counseling

## Abstract

Celiac disease is emerging in India and has become a public health problem. Almost 6–8 million Indians are estimated to have celiac disease. While there is a large pool of patients with celiac disease in India, until now, only a fraction of them have been diagnosed. With increasing awareness about celiac disease amongst health care providers and the general population, a massive increase in the number of patients with celiac disease is expected now and in the subsequent decade in India. While the number of patients with celiac disease is increasing, the country’s preparedness towards the emerging epidemic of this disease is minimal. There are a number of issues, which requires urgent attention. Some of the key issues include increased awareness amongst health care professionals and the general public about the disease and its management, team-based management of patients with celiac disease, proper counseling and supervision of patients, training of dietitians in the management of patients with celiac disease, industrial production of reliable and affordable gluten-free food, and food labeling for gluten contents.

## 1. Introduction

Celiac disease (CeD) is an immune-mediated enteropathy caused by exposure to gluten in genetically susceptible individuals [1,2]. Once thought to be a rare disease and believed to occur only in Western Europe, CeD is now a global disease and affects almost 0.6%–1% of the world’s population [3,4]. After Europe, America (both North and South), and the Middle East, it is now emerging in the East, including many Asian countries [5,6,7,8,9,10,11,12,13]. Also, once thought to be a disease of children and therefore to be managed mainly by pediatricians; CeD is now known to affect all the age groups including the elderly [14]. While CeD is emerging, the level of the awareness it too low amongst health care professionals, even amongst those most closely involved such as general physicians, family physicians, internists, gastroenterologist and pathologists [7,9].

## 2. Emergence of CeD in India

An increase in number of patients with CeD has been observed from many centers in India including ours [15,16]. Furthermore, two community based prevalence studies have been reported, both from the Northern part of India. In the first report from Ludhiana (Punjab), a questionnaire based survey of 4347 school children (3–17 years), Sood *et al.* [17] reported prevalence of CeD to be 1 in 310. In another community-based study including 10,488 subjects, both children and adults, we reported the prevalence of CeD in the Northern part of India to be 1.04% (1 in 96) and the prevalence of seropositivity (anti-tTG ab) to be 1.44% (1 in 69) [18]. Based on these two general population based studies, 5–8 millions of Indians are expected to have CeD. Of such a large pool of patients, only a fraction has been diagnosed to have CeD. The results of these studies suggest that CeD is a much greater problem in India than has been previously thought [18].

At present, only those with the most typical manifestations of CeD come to clinical attention and are ultimately diagnosed to have CeD. Now screening programs within populations indicate that celiac disease is under-diagnosed and what we detect clinically represents only the tip of the iceberg. With an appreciation of the existence of an iceberg of CeD in any society, and with increased awareness of CeD, patients with even milder symptoms are likely to be diagnosed in the coming years [19,20]. Currently, most celiac specific serology ELISA kits in India are imported from Europe. Their diagnostic cut-off values of antibody concentrations are based on Caucasian population data. With the difference and diversity in gluten ingestion, the cut-off values for a positive test in India may not be similar to those reported in the Caucasians.

## 3. Evolution of Dietary Management of CeD

Dietary management was a mainstay of treatment of CeD even in the early part of the 20th century [21]. During 1930s, clinical improvement was observed with several differing diets including an oyster diet suggested by Gee and the banana diet popularized by Haas [22]. Stools of such patients were quite greasy and worsening of their diarrhea after a carbohydrate diet led to another dietary approaches such as reduction or almost complete elimination of dietary fat or carbohydrates. A remarkable observation by a Dutch pediatrician, Willem Dicke [23], gave the birth to an idea from listening to one of his child patients’ mothers. The mother of the patient told Willem Dicke that her child used to become better if he did not eat porridge. From a clinical observation of one child and through years of clinical questioning and dietary therapy, he concluded that wheat was the toxic agent leading to CeD [23,24]. Toward the end of World War II, the so-called “winter of starvation” when even bread was not available in Holland; children with CeD paradoxically improved even though they were consuming a starvation diet (almost devoid of wheat products). When bread was airdropped in Holland, deterioration was noticed in these children [23]. Such an observation further strengthened the idea that some of the ingredients of wheat were the toxic agents for CeD.

## 4. What Is Gluten?

The protein content of wheat varies between 8% and 17%, depending on the genetic make-up and external factors associated with the crop. When wheat flour is washed with water, the insoluble protein fraction forms a viscoelastic protein mass, called gluten. Gluten, which comprises roughly 78% to 85% of the total wheat protein, is a very large complex mainly composed of polymeric (multiple polypeptide chains linked by disulphide bonds and monomeric (single-chain polypeptides) proteins. Gluten plays a key role in determining the unique-baking quality of wheat by conferring water absorption capacity, cohesively, viscosity and elasticity on dough [25,26,27].

Gluten is classified into two main fractions according to their solubility in aqueous alcohols: the fraction which is soluble in aqueous alcohol is gliadin and those insoluble are called glutenins. Both the fractions consist of numerous, closely related polypeptides that are rich in glutamine and proline amino acids. Gliadins are mainly monomeric proteins with molecular weights around 28,000–55,000 and they are further subclassified as α/β-, γ- and ω-type according to differences in their primary structures. Glutenins consist of glutenin subunits of high (*M*_W_ 67,000–88,000) or low molecular weight (*M*_W_ 32,000–35,000) that are connected by intermolecular disulphide bonds. Non-covalent bonds such as hydrogen bonds, ionic bonds and hydrophobic bonds bind gliadins and glutenins, which provide structure and physical properties of the gluten. Glutenins confer elasticity, while gliadins mainly confer viscosity and extensibility to the gluten complex [25,26,27].

Gliadins and glutenins contain domains with numerous repetitive sequences rich in these amino acids [28]. The gliadins have high proline and glutamine content and humans inherently lack endopeptidases to cleave bonds between proline and glutamines. The incomplete digestion of gliadin by digestive tract enzymes leads to the generation of many polypeptides, which are immunogenic to patients genetically susceptible to CeD.

## 5. What Is a Gluten Free Diet (GFD)?

The absence of gluten in natural and processed foods represents a key aspect of the GFD (gluten free diet). The current Codex Standard for gluten free foods was adopted by the Codex Alimentarius Commission of the World Health Organization (Geneva, Switzerland) and by the Food and Agricultural Organization (Rome, Italy) in 1976 and amended in 1983. In 2000, the Codex Alimentarius Commission of the World Health Organization and the Food and Agriculture Organization described gluten free foods with a gluten level not exceeding 20 ppm and consisting of, or made only from ingredients which do not contain any prolamines from wheat or any Triticum species, such as spelt, kamut or durum wheat, rye, barley, oats, or their crossbred varieties [29,30].

Gluten intake varies from population to population and depends upon dietary practices. In a double blind, placebo controlled prospective study; Catassi *et al.* [31] demonstrated that an intake of as little as 50 mg of gluten per day for 3 months was sufficient to cause a significant decrease in the intestinal mucosal mucosal villous height/crypt depth ratio. Furthermore, a daily intake of gluten of lower than 10 mg is unlikely to produce significant histological abnormalities [32]. The GFD with the threshold at less than 20 ppm of gluten ensures an intake of less than 50 mg/day and provides a sufficient safety margin [33].

## 6. Indian Dietary Habits and Gluten in the Indian Diet

India is a country of diversity in terms of culture, language, living standards and dietary practices. Until a few decades ago, Indians used to prefer natural foods to refined foods. In general, Indian families cook most of their meals at home on a daily basis and eat freshly cooked warm food. Most Indians do not prefer frozen or packaged food and tend to prepare their food from fresh ingredients. Indians are also less likely to visit restaurants. The family and social bond is very strong and they like to have food together with family members. Most of the meals contain a combination of cereals, pulses, spices and vegetables. Those who can afford, also uses dairy products, fruits and non-vegetarian food.

The “Green Revolution” in 1970s and onwards, promoted record grain promoted and ensured self-sufficiency in cereal grains [34,35]. During the past two decades, a shift from traditional to modern technologies, globalization, industrialization, constant travels across the world, and a fast growing economy led to the use of processed and fast foods at least in the urbanized part of India. While on one hand, there is poverty and hunger causing under-nutrition and its related disorders; a substantial increase in the intake of fast food is on the other hand leading to over-nutrition related disorders such as obesity and diabetes [36,37,38]. Therefore, the effects of malabsorption secondary to CeD could be severe in those who already have poor nutritional status due tounderlying poverty and ignorance.

Gluten intake varies from population to population and depends upon dietary practices. Wheat is the staple cereal in the northern part of India and flat bread made from wheat flour is the one of the most important constituents of almost every meal. In the southern and northeastern part of India, rice is a staple cereal. A typical North Indian diet, where flat bread is the usual meal, contains about 25–30 g of gluten per day; whereas average gluten intake in the West varies from 10 to 20 g/day [39]. 

## 7. Barriers in Maintaining a Strict GFD in India

There are many barriers to maintenance of a GFD; some of them are universal and some of them are unique to Indian patients with CeD. Patients with CeD are challenged with barriers in maintenance of a strict GFD because of factors such as inadequate information and education about the disease, food contamination, and inadequate/no food labeling on the packaged food items [40,41].

Because of viscoelastic properties, as discussed earlier, gluten is used extensively in the food industry. In fact, efforts have been made to increase the gluten content of the wheat and globally the gluten industry is very big. It may be surprising to know that gluten is present in daily use items such as lipsticks, postage stamps, beer, ice-creams, sweets, confectionary foods spreads and seasonings, soups and sauces, malted beverages and many more [42]. Due to the lack of gluten labeling on food items in India, it is difficult for anyone to know if a particular food product is gluten free or not [43,44].

Contamination of food with gluten is another concern. The contamination of food with gluten can occur: during milling if the same mill is used without proper cleaning for grinding gluten-containing and gluten-free grains; at the grocery store, if the same spatula is used to pick gluten containing and gluten free grains/flours; at factories producing commercial food products if same production line and equipment are used for both gluten-containing and GF (gluten free) food products; during preparation of commercial food products where gluten is added as fillers, stabilizing agents or processing aids such as thickener in soups, canned vegetables and other processed foods; at home, if the same utensils are used for storing, cooking and handling (rolling pin, surface griddle, dusters and oil for frying) gluten-containing and GF cereal flours and products; and while eating out, if addition of thickeners fillers/binders had been used which may unintentionally contain gluten.

Successful management of CeD requires a team approach, including patient, family, physicians, and dietitian. After a diagnosis is made, all the patients should be referred to a dietician for nutritional assessment, diet education, meal planning, and assistance with the social and emotional adaptation to the GF lifestyle. A delay in referral, or no referral at all, increases the likelihood of the patient obtaining inaccurate information from the Internet, health food stores, alternative health practitioners, family, friends, and other sources, which may be outdated, inaccurate, and/or conflicting. This results in confusion, frustration, and insufficient knowledge regarding CeD and the GFD. The poorly informed patients might unnecessarily restrict certain foods, thus limiting the variety and nutritional quality of their diet [45].

Adherence to GFD is the most critical factor for remission of CeD. Adherence to GFD is complex and is influenced by knowledge, country or region of residence, availability of GF food, determination, and social support [46,47,48,49]. Furthermore, attitude and behaviors of significant others such as family, teachers and friends also affect the decision to comply with GFD. Compliance to GFD is best maintained when residing at home; and eating out with friends or at school are the most difficult places to comply with GFD [40]. Lack of background awareness about the CeD and its strict dietary restriction in the community creates a problem for the patient and the family of patients with CeD [1,40]. Furthermore, it has been observed that girls face compliance issues after marriage. The compliance to GFD is better if CeD is diagnosed in their early part of life compared to those in whom the diagnosis is made later in life. Patients having minimal symptoms or those diagnosed on screening comply less well than those who have overt symptoms [50,51]. As the patient grows from childhood to adulthood and become asymptomatic, the compliance to GFD gets worse. Non-appearance of acute symptoms after inadvertent or deliberate ingestion of gluten might make the patient more confident and induce them to try gluten at other occasions also. Many patients in remission might try gluten to see what happens to them with gluten intake [46,47,48,49,50].

### Non-Availability of GF (Gluten Free) Food Products

Because of the perception that CeD is uncommon in India and a low absolute number of patients with CeD in India, the need for making a GFD available has not been perceived. The diagnosis of CeD presently is limited to only at the secondary and tertiary care centers and often more so in the northern part of India. There is some small-scale production of GFD (mainly flour and biscuits) food in certain sectors in the northern part of India, which relies upon mixing of non-wheat and non-barley cereals. There is a possibility of contamination at various levels such as during harvesting, storage and packaging of grain bags by the farmers. In fact all of these should be taken into consideration before the grains are used for making gluten free flour. Whichever gluten free flour/products are available do not undergo rigorous quality check for their gluten contents. The production of GFD in India till now has been small-scale industry based only, and the varieties and choices of GFD are also extremely limited.

Availability of GF foods is a factor, which determines compliance to GFD. Furthermore, difficulty in obtaining GF food also interrupts compliance to GFD. Even in countries where CeD is common, 10%–15% patients report difficulty in getting a continuous supply of GF food [52]. Non-availability of GFD outside their home environment restricts their travel, occupation and profession.

GF food items are considerably more expensive than regular gluten-containing food [53]. A nutritionally-balanced GF market basket, based on foods typically consumed by the Scottish population, costs more than a standard market basket [54]. Therefore, patients with CeD have additional financial requirement for purchasing of GFD for their living.

## 8. Measures to Break the Barriers to GFD

### 8.1. Patient Education and Awareness

The management of CeD is unique and different from the treatment of other medical or surgical diseases. At present, life-long and complete avoidance of gluten from the diet is the most effective treatment of CeD. While prescribing GFD is easy; the key to the success is the dietary counseling by a dietician and maintenance of compliance by the patient [44,46,47,48]. Like any other chronic disease management, education of the patients and their families about the disease and dietary restrictions is of immense importance. It is generally not possible to explain everything about the dietary restrictions in one visit from the nutrition specialist. The understanding and maintenance of GFD requires consistent supervision and guidance, which is best provided to the patients and families on multiple visits. At every visit to the hospital/clinic, the level of compliance to gluten avoidance should be checked and appropriate guidance should be provided.

### 8.2. Training of Nutritionist in CeD Management and Counseling of Patients

The dietary councilor should have sufficient knowledge about the GF food and food products. It is not only about prescribing GFD but it is essential to provide for the patient a specific well-balanced diet. The dietitian is the most qualified health care professional to provide nutrition therapy. Dietitians have extensive academic and practical experience including in-depth knowledge of nutrition, nutritional needs, nutrition composition and food preparation information and educational factors that affect food and nutrition behavior of people. They are also skilled to translate scientific information into laymen’s terms and assist individuals in gaining knowledge, self-understanding, improved decision making, and behavioral changes. Although other health care professionals can disseminate nutrition advice, they do not have the training in nutrition sciences and food composition to be able to translate complex medical nutrition concepts and issues into attainable dietary changes [42,44,46,47,48].

### 8.3. Celiac Disease Support Groups

Celiac disease support groups provides a platform for patients to discuss their problems amongst themselves and learn from each other, provides information about GF products and their availability, but also can act as an advocacy for gluten labeling and other issues to the Government and regulatory bodies [55,56,57]. Furthermore, it has been observed that the adherence to a GFD increases when individuals are members of a patient support group [55,56,57,58,59].

### 8.4. Development of Reliable GFD at a Large Scale

While the number of patients with CeD is small at present in India, the absolute number of patients is rising day after day. As awareness about the disease increase, there is a likelihood of an exponential increase in the number of patients with CeD and hence the demand of GFD is likely to increase in the near future. Therefore, there is need for large-scale industrial level production of reliable and affordable GF food, including choices of food products ranging from snacks, flour, sweets, ice-creams and ready-to-eat packets.

All GF food products should be tested for their quality before releasing for patient’s use. In fact, there should be certified gluten check laboratories where food items may be checked for their gluten contents. This is an important step to ensure quality of food for its gluten content until the gluten labeling legislation is enforced.

These foods, which are available at present in India, are not labeled for their gluten content. As mentioned above, gluten is used extensively in the food industry. Furthermore, even a small amount of gluten can maintain the disease activity [31,32,33]. It is therefore essential that food products, which are available, should be labeled for gluten content.

## 9. Combining Diabetes and Gluten Free Dietary Management Guidelines

Eight to fifteen percent of patients with CeD have type I diabetes [58,59]. Planning of a dietary management for such patients is a challenge for endocrinologists and dietitians. Disclosure of dietary restrictions with a diagnosis of CeD in a patient with type I diabetes may be received as a shock to the patient and the family. The objectives of diet planning for such patients are to provide a balanced nutritive diet with restrictions posed by diabetes and CeD. A healthy eating plan for diabetes should always be individualized based on the patient’s need and metabolic outcome goals (HbA1C, weight/height, lipid profile, blood pressure, *etc.*) [60]. It is therefore essential that rather than making two dietary plans for control of CeD and diabetes, the dietitian should make one unified dietary plan for both the co-existing conditions.

## 10. Conclusions

CeD is a public health problem and 6–8 million Indians are estimated to have CeD. While there is a large pool of patients with CeD in India, only a fraction of them are currently diagnosed. With increasing awareness about CeD amongst health care providers and the general population, a massive increase in the number of patients with CeD is expected in the present and subsequent decade in India. While the number of patients with CeD is increasing, the country’s preparedness towards this disease management is extremely minimal. There are a number of issues, which require urgent attention. Some of the key issues include team-based management of patients with CeD, proper counseling and supervision of patients, training of dietitians in the management of patients with CeD, industrial production of reliable and affordable GF food, food labeling for gluten contents and increase in awareness amongst health care professionals and the general public about the disease and its management.
